# miRNAs affect the development of hepatocellular carcinoma via dysregulation of their biogenesis and expression

**DOI:** 10.1186/s12964-014-0045-y

**Published:** 2014-07-11

**Authors:** Rui Chu, Guangquan Mo, Zhijun Duan, Mei Huang, Jiuyang Chang, Xiaodong Li, Pixu Liu

**Affiliations:** 1 The First Affiliated Hospital Collaborative Innovation Center of Oncology-Institute of Cancer Stem Cell, Dalian Medical University, Dalian 116011, China; 2 Department of Gastroenterology, The First Affiliated Hospital of Dalian Medical University, Dalian 116011, China; 3 Institute of Cancer Stem Cell, Dalian Medical University, Cancer Center, Dalian 116044, China; 4 Department of Anesthesiology, Dalian Medical University, Dalian 116044, China

**Keywords:** miRNA, miRNA biogenesis, Hepatocellular carcinoma, Molecular mechanism

## Abstract

The pathogenesis of hepatocellular carcinoma (HCC) is not fully understood, which has affected the early diagnosis and treatment of HCC and the survival time of patients. MicroRNAs (miRNAs) are a class of evolutionarily conserved small, non-coding RNAs, which regulate the expression of various genes post-transcriptionally. Emerging evidence indicates that the key enzymes involved in the miRNA biosynthesis pathway and some tumor-specific miRNAs are widely deregulated or upregulated in HCC and closely associated with the occurrence and development of various cancers, including HCC. Early studies have shown that miRNAs have critical roles in HCC progression by targeting many critical protein-coding genes, thereby contributing to the promotion of cell proliferation; the avoidance of apoptosis, inducing via angiogenesis; and the activation of invasion and metastasis pathways. Experimental data indicate that discovery of increasing numbers of aberrantly expressed miRNAs has opened up a new field for investigating the molecular mechanism of HCC progression. In this review, we describe the current knowledge about the roles and validated targets of miRNAs in the above pathways that are known to be hallmarks of HCC, and we also describe the influence of genetic variations in miRNA biosynthesis and genes.

## Introduction

Hepatocellular carcinoma (HCC) is the fifth most commonly diagnosed cancer worldwide but the third leading cause of cancer-related death around the world. Moreover, the incidence of HCC is over 50 million every year [[1]]. Studies have gradually elucidated the pathogenesis of HCC in recent years. However, the early diagnosis and treatment of HCC in clinics are still quite challenging. Epidemiologic studies indicate that the major risk factor for HCC is chronic hepatitis virus infections, mainly the hepatitis B virus (HBV) and the hepatitis C virus (HCV); other risk factors include exposure to certain chemicals, intake of large amounts of alcohol, some inherited metabolic diseases and similar factors [[2]]. Although the etiology of HCC is relatively clear, the exact pathogenesis and pathways of HCC are not fully understood. In terms of disease processes, HCC develops from chronic diffuse liver disease and cirrhosis. Recent research developments on the underlying pathogenesis of HCC indicate that its incidence is mainly caused by repeated repair and regeneration, inflammation and oxidative DNA damage to liver cells [[3]]. Of these, the mechanism by which microRNAs (miRNAs) regulate HCC development has recently become a focus of research in molecular biology. Increasing evidence indicates that miRNAs are expected to become new diagnostic markers and therapeutic targets of HCC.

miRNAs are small, evolutionarily conserved, single-stranded RNA molecules that are approximately 21–24 nucleotides in length. miRNAs regulate gene expression by binding to specific mRNA targets and promoting their degradation and/or translational inhibition [[4]]. As regulators of gene expression, miRNAs fine-tune a variety of essential cellular processes, including cell growth, differentiation, metabolism and apoptosis [[5]]. The vast majority of miRNAs can bind to their target mRNAs through 3’UTR interactions [[6]]. For the mechanism of tumor formation, miRNAs can play roles of oncogenes or tumor suppressor genes because of the combination of different target mRNAs. Abnormal activation and inactivation of oncogenes and tumor suppressor genes are important factors leading to malignancy (including HCC). Clarifying the molecular mechanisms of HCC could provide a basis for HCC risk assessment, early diagnosis, effective treatment and intervention. In this review, we will summarize the influence of the abnormal biosynthesis of miRNAs and the aberrant expression of miRNAs on the cell cycle of tumor cells, angiogenesis, the activation of invasion and metastasis and the occurrence and development of HCC. Understanding the molecular pathogenesis of HCC can provide evidence for assessing predisposing factors, early diagnosis, treatment and intervention for HCC.

### The basics of miRNA biogenesis

The synthesis of miRNA mainly consists of two steps, including nuclear synthesis within and outside the nucleus. First, RNA polymerase II acts on the miRNA coding region in the nucleus, and the coding region is transcribed to primary miRNA (pri-miRNA) that contains hundreds of thousands of nucleotides. Subsequently, pri-miRNAs are processed to an miRNA precursor (pre-miRNA) in the nucleus by the microprocessor complex, which consists of the nuclease Drosha and DiGeorge syndrome critical region gene 8 (DGCR8). Pre-miRNAs are approximately 60 to 70 nucleotides in length with a stem-loop structure [[7]]. Then, pre-miRNAs are transported to the outside of the nucleus with the help of the RAS-related nuclear protein with bound GTP (RAN-GTP)-dependent transporter exportin-5 (XPO-5). Pre-miRNAs are released from the Drosha-DGCR8 microprocessor complex in the nucleus, which has a high concentration of RAN-GTP, and are transferred to the cytoplasm along with XPO-5. Pre-miRNAs are released in the cytoplasm, where the concentration of RAN-GTP is low. In the cytoplasm, pre-miRNAs undergo additional cleavage by the RNase III endoribonuclease Dicer. The result of this process is the formation of an incomplete matching duplex approximately 21 to 24 nucleotides in length composed of the mature miRNA, which is consequently connected to the RNA-induced silencing complex (RISC) and miRNA*, which is degraded after separation from the complex [[8]]. Finally, with the help of helicases such as Gemin3 or RCK/p54, mature single-stranded miRNAs are generated. The TAR RNA binding protein or TARBP2 (TRBP), Argonaute 2 (Ago2) and Dicer are involved in the formation of the RISC loading complex (RLC), which can facilitate the binding of the mature miRNA to RISC, where it mediates gene silencing either by translational inhibition or by promoting the degradation of target mRNAs [[9]]. RISC recognizes the target mRNA by imperfect pairing with mRNA 3’ untranslated regions (3’UTR) and perfect pairing with the target mRNA. This recognition results in mRNA degradation and thus the degradation or translation inhibition of the target genes [[10]]. Gemin4 is one of important components that contributes to both miRNA processing and target gene silencing [[11]].

### Role of the aberrant expression of key enzymes involved in the miRNA biosynthesis pathway in the occurrence and development of hepatocellular carcinoma

**Figure 1:**
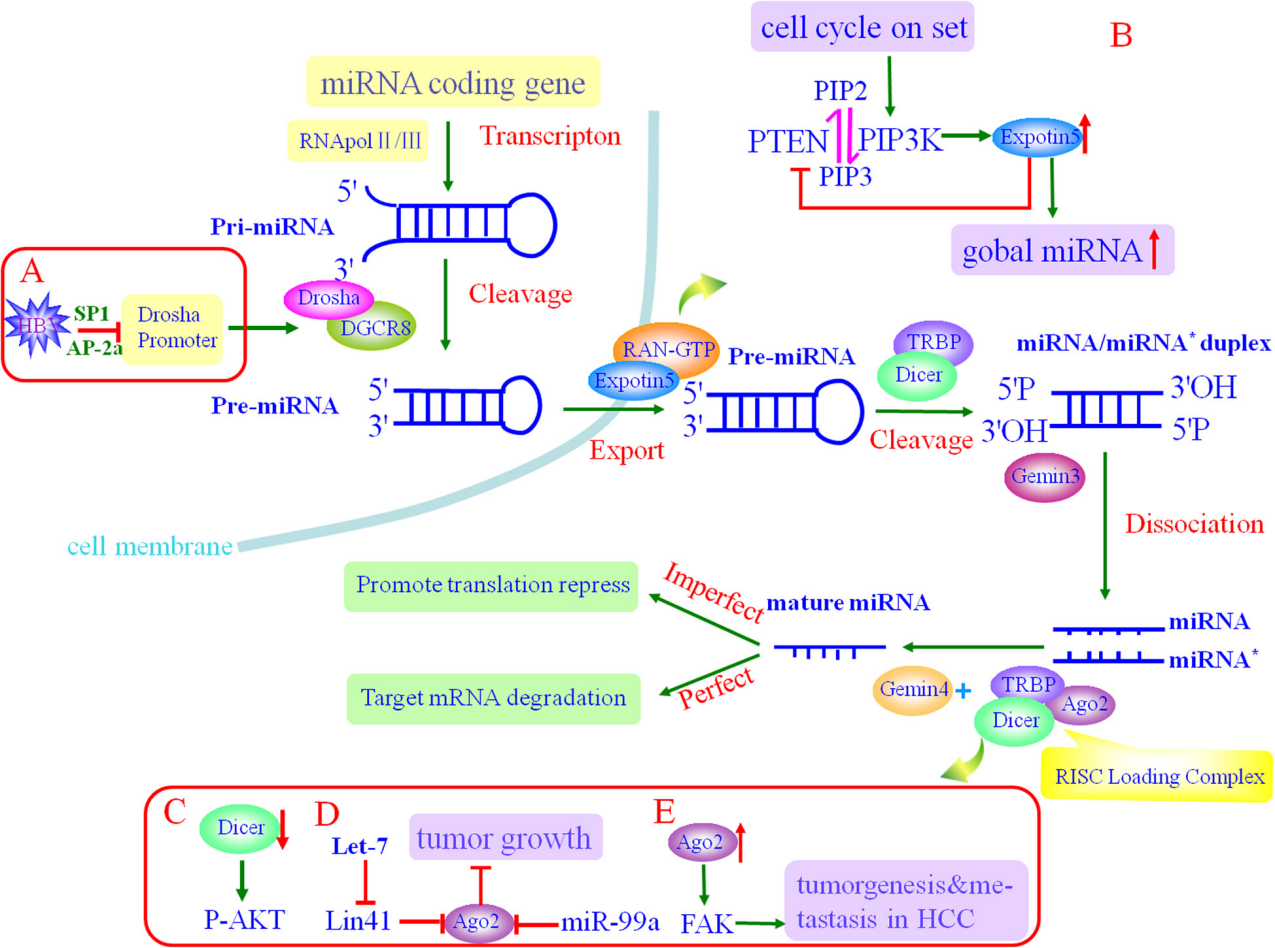
**Schematic representation of the biogenesis pathway of microRNAs and the aberrant expression of key enzymes involved in this pathway in HCC.** First, miRNAs are transcribed by RNA pol II/III from the miRNA coding gene to pri-miRNAs, and soon afterwards microprocessor complex recognizes pri-miRNA to generate pre-miRNA. XPO-5 with its cofactor RAN-GTP mediates the nuclear export of pre-miRNA. Finally, the RLC loading complex assists in the binding of mature miRNA to RISC to mediate gene silencing. However, in HCC, the expression of some key enzymes involved in this pathway appears to be aberrant, and this aberrant expression results in an abnormal microRNA biogenesis pathway (shown in A/B/C/D/E).

As the key upstream regulatory factors of miRNAs, the key enzymes involved in the miRNA biosynthesis pathway include Drosha, DGCR8, XPO-5, RAN, Dicer, TRBP, AGO1, AGO2, Gemin4 and Gemin3. The aberrant expression of these enzymes can change a series of downstream miRNAs and factors, which promote or inhibit tumors to interact with each other. Next, the role of some key enzymes in the miRNA biosynthesis pathway and their abnormal expression will be introduced in the occurrence and development of hepatocellular carcinoma (Figure 1).

### Aberrant key enzymes involved in the regulation of the processing step from pri-miRNA to pre-miRNA

Research indicates that Drosha and DGCR8 are the key enzymes involved in the regulation of the processing step from pri-miRNA to pre-miRNA in many types of tumor cells. However, the pri-miRNA to pre-miRNA processing of some miRNAs is relatively independent of the complex of purified DGCR8 and Drosha [[12]]. Drosha and DGCR8 are frequently over-expressed in HCC. Angela M. Liu and colleagues recently showed that of the genes involved in the biosynthesis of miRNAs, DROSHA is the most differentially expressed in HBV-associated HCC [[13]]. Moreover, aberrant expression of Drosha can be observed in ovarian cancer, cervical cancer and breast cancer, suggesting that Drosha is involved in many tumors [[14]]. In addition, the activity of Drosha can be inhibited by HBV. Min Ren and colleagues found that HBV could inhibit the promoter activity of Drosha via HBx and thereby could downregulate Drosha expression; the transcription factors SP1 and AP-2a might assist in this downregulation [[15]].

### Aberrant key enzymes involved in controlling the nuclear export of pre-miRNA

The nuclear export of pre-miRNAs mostly relies on XPO-5, whose damage in tumor cells may result in global downregulation of mature miRNAs. Pre-miRNAs are retained in the nucleus in various types of human cancer; thus, a gene defect in the nuclear transporter of pre-miRNAs may be associated with the occurrence of human tumors [[16]]. XPO5-inactivating mutations are always observed in many human tumors, indicating that XPO5 is a candidate haplo-insufficient tumor-suppressor gene [[17]]. During cell cycle entry, the general elevation of miRNAs is important for controlling gene expression. Yuka W and colleagues showed that XPO5 is induced by a PI3K-dependent post-transcriptional mechanism and that the suppression of XPO5 can interfere with the general elevation of miRNA, leading to dysfunctional cell proliferation related to the G1/S transition. However, XPO5 is involved in the export of not only pre-miRNAs but also other non-miRNA molecules [[18]]. Although research has demonstrated the elevation of primary miRNAs that are likely to target key cyclin, there is still possibility of non-miRNA XPO5 targets of cell cycle control. Verification of the latter possibility requires further study [[17]].

### Aberrant key enzymes involved in the regulation of the processing step from pre-miRNA to mature miRNA

Dicer is responsible for the dicing and maturation of miRNA. Most miRNAs are Dicer dependent. However, some scholars have reported that the dicing and maturation of miR-451 are dependent on Ago2, not Dicer [[19],[20]]. Dicer is coded by the human Dicerl gene, and the latter acts as a haplo-insufficiency tumor suppressor gene [[21]]. In addition, some studies have demonstrated that Dicerl is the susceptibility gene of familial pleuropulmonary blastoma by family-based linkage analysis and gene mutation detection [[22]], and in a mouse model, the monoallelic Dicerl-knockout may lead to retinoblastoma [[21]]. All these findings indicate that Dicerl mutation can increase tumor susceptibility.

Dicer is downregulated in most carcinomas, including HCC. The Dicer level is significantly lower in HCC than in adjacent non-neoplastic tissues [[23]]. Sekine et al. found increased expression of growth promotion genes and the embryonic stage of specific genes in the liver cells of Dicerl-knockout mice. The Dicer1 knockout led to both increased hepatocyte proliferation and apoptosis and a change in blood vessel formation and remodeling [[24],[25]]. More than 60% of the Dicer1-deficient mice developed HCC derived from residual Dicer1-deficient hepatocytes at 1 year of age. These findings demonstrate that Dicer may play a key role during the process of hepatocarcinogenesis. Han et al. used the shRNA of adenoviral vector to silence the expression of Dicer in tumor cells. They found that the ability of tumor cells to proliferate and invade greatly improved, which was related to the activation of the p-AKT gene and the increased expression of cyclin A, PCNA and the invasive proteins MMP-2 and MMP-9. These results showed that the downregulation of Dicer in tumor cells can promote tumor development via an indirect mechanism. However, the upregulation of Dicer expression still occurs in a handful of tumors. For example, the expression level of Dicer mRNA in patients with stage 3 colorectal cancer is higher than those at stage 2 [[26]]; in bronchoalveolar carcinoma and lung adenocarcinoma, Dicer mRNA is upregulated [[27]]. Therefore, a low expression level of Dicer may promote the progress of most tumors that are characterized by higher malignancy and poorer prognosis. However, the opposite conclusions have been reported in the study of colorectal cancer, bronchoalveolar carcinoma and lung adenocarcinoma, which suggests that the carcinogenic mechanism of Dicer is tumor specific. The reason of this radical difference is unknown.

Another key enzyme involved in the regulation of the processing step from pre-miRNA to mature miRNA is Ago2. Over-expression of Ago2 markedly reduces HCC growth. The function of Ago2 can be adjusted by miRNAs. For example, the tumor suppressor miRNA let-7 could target Lin-41, whose function is to regulate the ubiquitylation and degradation of Ago2; this change thus leads to a high tumor grade and a high tumor stage. Lin-41 can be a predictor of early intrahepatic recurrence. Over-expression of Lin-41 can suppress the expression of the Ago1 and Ago2 of RISC and this suppression can promote the growth of tumor cells. These findings indicate that Lin-41 leads to cancer by suppressing RISC [[28]]. In addition, miR-99a directly regulates Ago2 through translational repression in HCC. Downregulation of miR-199a and upregulation of Ago2 are also inversely associated in HCC [[29]]. These studies provide potential strategies for HCC therapy by regulating Ago2 to activate RISC or reintroduction of miRNA suppressors.

### miRNA and genetic predisposition for HCC

There are genetic differences in the DNA sequences of various individuals, and the chief difference occurs as a single-nucleotide polymorphism (SNP). The severity of disease and the way our body responds to treatment are also manifestations of genetic variations. Genetic variations, mostly SNPs within miRNA-processing genes, miRNA sequences and miRNA-binding sites, have been found to be associated with various types of cancers. These three types of SNPs could affect cancer risk [[30]]. In recent years, increasing research has indicated a close relationship between SNPs in microRNA regulatory genes and the genetic susceptibility to primary liver cancer [[31]]. SNPs may affect the activity of key enzymes involved in the miRNA biosynthesis pathway. For example, one group found that the genotypes of CT/CC in the rs1057035 locus of the DICER gene could decrease the risk of HCC significantly while the AG/GG in the rs3803012 locus of the RAN gene might increase the risk of HCC. Thus, the combined effects of multi-gene alleles and multi-locus genotypes might have a synergistic role in the carcinogenesis of liver cancer [[32]]. In addition, the genotype of AA in the rs11077 locus of the XPO5 gene might be genotype susceptible to the worse survival in HCC patients [[33]]. These facts indicated that the SNP of key enzymes involved in the miRNA biosynthesis pathway might play a critical role in tumorigenesis. Furthermore, these SNP-related studies must be verified for HCC. Given the importance of SNP in miRNA function, SNPs are thought to affect the susceptibility of tumor sequentially. The TC/CC genotypes in rs4938723 in the promoter region of pri-miR-34b/c were investigated and shown to be associated with significantly increased susceptibility and risks in HCC compared with the wild-type TT patients [[34]]. However, the patients with CT and CC genotypes of the rs11614913 SNP in mature miR-196a2 might have altered susceptibility and progression of HCC and also have a higher risk of developing HCC [[35]]. Similarly, a study detected that the rs999885 AG/GG genotypes located in the miR-106b-25 cluster sequence were apparently associated with an increased HCC risk compared with that in the AA genotype carriers [[36]]. SNPs located at the 3’ untranslated regions (3’UTR) of genes might modulate the expression of target genes by influencing the stability and translation regulation of certain mRNAs. For example, in one meta-analysis, the miR-146a*C variant (rs2910164) and miR-196a-2*T (rs11614913) contained in the genes encoding miR-146a and miR-196a-2, respectively, were shown to decrease the risk and increase the susceptibility to HCC, respectively [[37]]. In summary, miRNA-related SNPs play a substantial role in cancer development (Table 1). However, we currently have little knowledge of the overall contribution of these types of SNPs to tumorigenesis. Therefore, additional research designed to study the relationship between cancer risks and miRNAs and their related SNPs in the whole genome is warranted.

**Table 1:** miRNA-related SNPs in hepatocellular carcinoma

**SNP**	**Correlation**	**Comparison model and** ***P*** **value**
miR-DICER rs1057035	Higher risk	CT/CC vs TT,OR = 0.79,95% CI = 0.64-0.96, *P* < 0.05
miR-RAN rs3803012	Lower risk	AG/GG vs AA,OR = 1.35,95% CI = 1.03-1.77, *P* < 0.05
miR-XPO5 rs11077	Worse survival	AA vs AC/CC,OR = 0.395,95% CI = 0.167-0.933, *P* < 0.05
pri-miR-34b/c rs4938723	Higher susceptibility and risk	TC/ CC vs TT,OR = 1.580,95% CI = 1.029-2.426, *P* < 0.05
miR-196a2 rs11614913	Higher risk	CT/CC vs TT,OR = 1.784,95% CI = 1.082-2.944, *P* < 0.05
miR-106b-25 rs999885	Higher risk	AG/GG vs AA,OR = 1.250,95% CI = 1.060-1.470, *P* < 0.05
miR-146a rs2910164	Lower risk	CC vs CG/GG,OR = 0.850,95% CI = 0.760-0.960, *P* < 0.05

### The regulation of miRNAs in HCC

Ten biological capabilities acquired during the multistep development of human neoplasms form the hallmarks of cancer [[38]]. Some of these hallmarks are actually regulated by miRNAs. The expression of aberrant miRNAs are included in the molecular pathways and biological functions altered in hepatocarcinogenesis. Furthermore, miRNAs play essential roles in the cell cycle, apoptosis, angiogenesis, metastasis and other important processes [[5]]. Understanding the biological roles and specific targets of miRNAs will open up new avenues for investigating crucial cancer-associated mechanisms in liver.

### Deregulated miRNAs involved in the cell cycle

**Figure 2:**
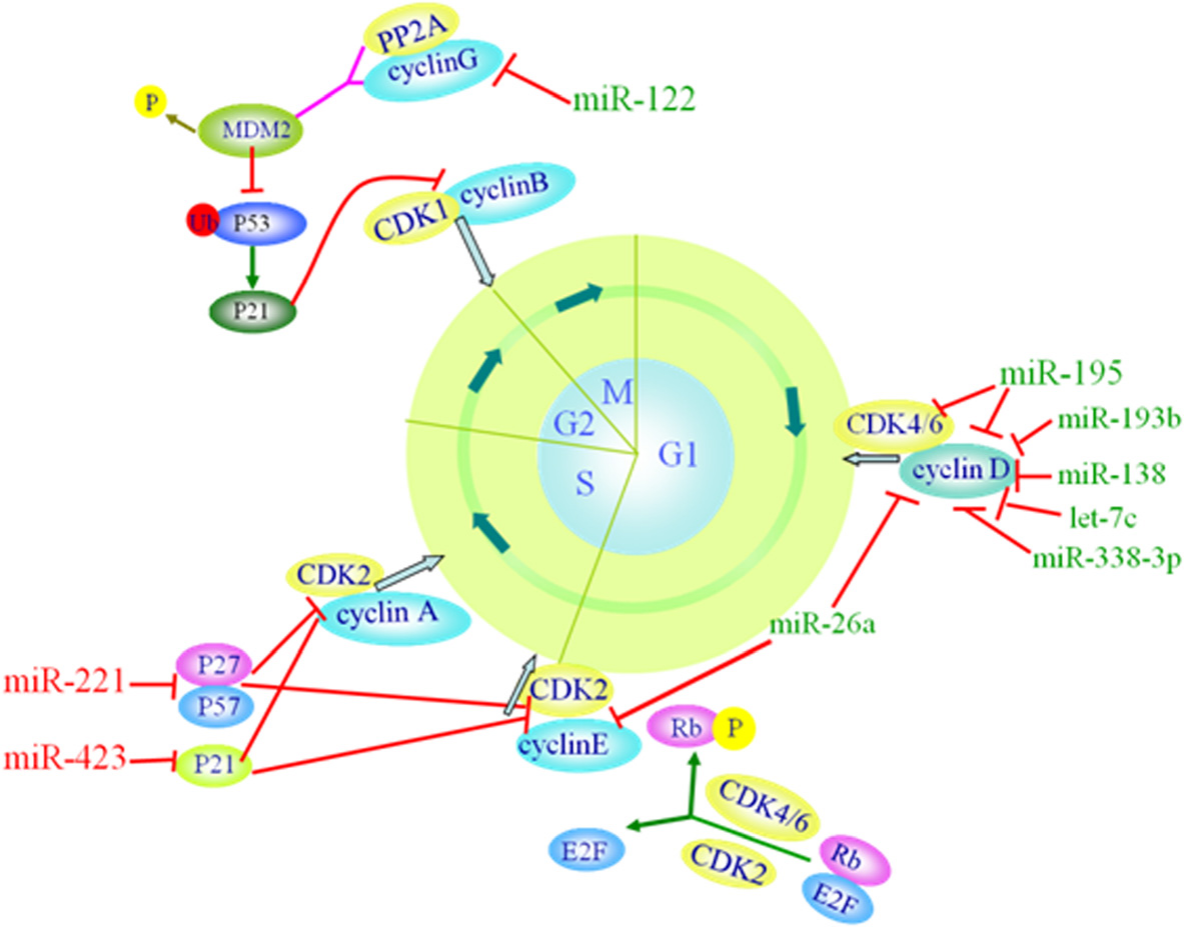
**Deregulated miRNAs in HCC affect cell cycle.** These miRNAs impact cell cycle progression by targeting cell growth regulators. The green miRNAs may promote the HCC cell cycle, and the red miRNAs may inhibit the HCC cell cycle.

Cell cycle dysregulation is an essential step in the initiation and development of human malignancies, including HCC. Accumulating evidence has shown that deregulated miRNAs may affect HCC cell proliferation through direct interaction with critical regulators of cell cycle machinery [[39]]. Such miRNA targets include cyclin, cyclin-dependent kinases (CDK), cyclin-dependent kinase inhibitors (CDKI) and other cell growth regulators (Figure 2).

Cyclin D, with the help of CDK4/6, is a crucial mediator of the G1 to S progression. Cyclin D is divided into 3 types, that is, cyclin D1, D2 and D3. The upregulation of cyclin D1 has been shown to promote the rapid cell growth of HCC [[40]]. Many miRNAs that target cyclin D1 are significantly downregulated in HCC. For example, miR-338-3p inhibits tumor cell proliferation and induces cell cycle arrest at the G1/S checkpoint by directly targeting cyclin D1 expression in HBV-positive liver cancer cells [[41]]. On the one hand, miR-26 dramatically inhibited HCC cell proliferation by inducing G1 arrest [[42]]. miR-26a could target interleukin-6 (IL-6), which stimulates the phosphorylation of signal transducer and activator of transcription 3 (Stat3); subsequently, the expression of Stat3 target genes, including cyclin D1, was significantly reduced. Thus, miR-26a might suppress the tumor growth of HCC cell through IL-6-Stat3 signaling [[43]]. On the other hand, miR-26a is also an inhibitor of the G1/S transition that directly targets cyclin D2 and E2 [[44]]. Moreover, other miRNAs affecting the expression of cyclin D1 include miR-193b, and miR-195 [[45],[46]]. Apart from the silencing of cyclin D1, miR-195 could also directly target elements that are both upstream (such as CDK6) and downstream (such as E2F3) of Rb, providing new insight into the aberrant Rb-inactivation frequently found in HCC [[45]]. In addition, Cyclin D3, a target of miR-138, was observed to be negatively associated with miR-138, which was downregulated in HCC tissues compared with adjacent non-tumor tissues [[40]].

The combination of cyclin B with CKD1 is the crucial checkpoint for starting the G2/M transition. miR-122 is a crucial miRNA that indirectly regulates the interaction of cyclin B and CKD1 to arrest the cell cycle in the G2/M phase. miR-122 is a highly abundant liver-specific miRNA and is frequently downregulated in liver cancers. It functions as a tumor suppressor by directly targeting several positive regulators of cell cycle progression that have been implicated in tumorigenesis, including SRF, Igf1R, ADAM 17, ADAM10, cyclin G1, Wnt1, Bcl-w and PI3CG [[47]-[51]]. As a proto-oncogene, cyclin G1 is closely related to cancer [[52]]. Cyclin G1 is over-expressed in HBV-infected liver cells, where decreased expression of miRNA-122 is frequently found [[51]]. Excessive cyclin G1 in association with PP2A B subunits promotes the dephosphorylation of Mdm-2, which is a repressor protein of p53. Then, it could inhibit p53 ubiquitination, which accelerates the degradation of p53. However, p53 decreases the expression of cyclin G1. This shows a negative feedback. This process is known to inhibit apoptosis and thus leads to tumors with unrestricted growth. Additionally, the increase of miR-122 in HCC could inhibit cyclin G1 and the cell cycle arrest in the G2/M phase, which could inhibit the proliferation of cancer cells [[53]]. p53 upregulates the expression of p21, which is known to competitively inhibit cyclin B-CDK1 complexes, leading to cell cycle arrest in the G2/M phase. Yanmin Xu et al. also reported that Bcl-W and cyclin G1 were the targets of miR-122 [[54]]. In addition, there are other studies showing that miRNA-122 could inhibit proliferation in many liver cancer cell lines (such as HepG2, Hep3B, Huh7 and PLC/PRF/5) [[47],[55]]. In short, both of these investigations show that miRNA-122 can inhibit hepatocarcinogenesis by directly targeting cyclin G1.

Remarkably, there are still some miRNAs that act as oncogenic miRNAs and target negative regulators of the G1/S transition of the mitotic cell cycle, such as CIP/KIP family members, and those miRNAs are upregulated in human HCC and promote the proliferation of HCC cells. For example, p21Cip1/Waf1 was revealed to be a downstream target of miR-423-3p, and it is another miRNA that could notably promote the cell cycle progression at the G1/S transition in HCC cells [[56]]. Another investigation revealed that CDKN1B/p27 and CDKN1C/p57 were downregulated in HCC cells as direct targets of miR-221; the upregulation of miR-221 can promote cell proliferation and increase the progression to S phase [[57]].

### Deregulated miRNAs in apoptosis

Apoptosis is a natural barrier to tumorigenesis and tumor progression. Cancer cells evolve to evade apoptosis to escape from the supervision of the body and to survive in the difficult tumor environment. The Bcl-2 family is composed of a series of pro-apoptotic members, such as Bax, Bak, Bid, Bim and Bmf, as well as anti-apoptotic members, such as Bcl-2, Bcl-XL, Bcl-W and Mcl-1 [[58]]. After receiving a death signal, the pro-apoptotic members undergo dephosphorylation and cleavage, resulting in their activation and translocation to mitochondria; then, apoptosis is initiated. All BH3-only proteins require multi-domain BH3 proteins (such as Bax and Bak) to help them perform their intrinsic pro-apoptotic activities. These anti-apoptotic members could bind to and suppress those pro-apoptotic members, which could lead to mitochondrial membrane permeability changes via the mitochondrion-mediated apoptotic pathway. This change can keep mitochondria from releasing a proapoptotic factor, that is, cytochrome C (cytC), to prevent or delay cell death [[58],[59]]. When released from mitochondria, cytC in the cytoplasm forms a complex with Apaf-1 and caspase 9 by way of cascade amplification and continues to activate caspase 3 and other downstream caspases, eventually leading to apoptosis [[60]]. A great majority of deregulated miRNAs are involved in mitochondrion-mediated apoptosis.

There are many miRNAs that target anti-apoptotic members of the Bcl-2 family. Most are significantly downregulated in HCC. For instance, miR-16 and miR-29 are downregulated in HepG2 cells, and one of their target genes is confirmed to be Bcl-2 [[61],[62]]. There are some other miRNAs whose target gene is Mcl-1. Apart from silencing of Bcl-2, miR-29 can also directly target Mcl-1 in mitochondrion-mediated apoptotic pathway [[62]]. In addition, miR-101, miR-193b, miR-125b, and let-7c, which are downregulated in HCC cells, might exert anti-apoptotic action via targeting Mcl-1 [[63]-[66]]. The downregulation of miR-125b was frequently observed in HCC, and miR-125b induced apoptosis by directly targeting Mcl-1 and Bcl-w; miR-125b could also indirectly suppress the levels of Mcl-1 and Bcl-xL by attenuating the IL-6/STAT3 signaling in cell lines derived from the liver [[65]]. In addition, let-7c and let-7g can combine with the 3'-untranslated region of their target mRNAs, leading to an obvious decrease in the expression of Bcl-xl, which could lead to anti-apoptotic tendencies [[66]]. However, miR-122, as a hepato-specific microRNA, was also detected, and it directly targets the binding site within the 3'-UTR of Bcl-w in HCC cells [[67]], which can inhibit the apoptosis of tumor cells.

However, some miRNAs that inhibit apoptosis by targeting pro-apoptotic members of the Bcl-2 family are significantly upregulated in HCC. For example, the consistent overexpression of miR-25 promotes cell proliferation in HCC; miR-25 targets the BH3-only protein Bim [[68]]. In addition, miR-221 inhibits apoptosis by targeting the proapoptotic BH3-only protein Bmf in HCC [[69]].

### Deregulated miRNAs in angiogenesis

The growth of a solid tumor must rely on continuous and extensive angiogenesis. Tumor angiogenesis principally relies on vascular endothelial growth factor (VEGF)-driven angiogenic responses, which lead to a dysfunctional vasculature [[70]]. Moreover, the invasion of endothelial cells (ECs) occurs after the release of matrix metalloproteinase 2 and 9 (MMP2/9), which destroy and degrade the basal membrane and extracellular matrix, finally resulting in the migration of ECs and the formation of neovessels [[71]]. Some of these genes involved in angiogenesis have been reported to be affected by the altered expression of miRNAs. The miRNAs involved in angiogenesis mainly target VEGF and MMP-2/9; furthermore, they are frequently downregulated in HCC. For instance, Bisheng Zhou and colleagues demonstrated that the miR-503 downregulation in HCC plays an anti-angiogenesis role in hepatocarcinogenesis by targeting VEGF directly [[72]]. miR-491 and miR-29b could also target MMP-2/9. For example, the inhibition of miR-491 in HepG2 cells increased the expression of MMP-2/9, which induces angiogenesis [[73]]. Another study revealed that the inhibition of MMP-2 could phenocopy the anti-angiogenesis and anti-invasion effects of miR-29b, whereas, the introduction of MMP-2 could attenuate the function of miR-29b in HCC cells [[74]]. However, many elements involved in angiogenesis have not been found to be targets of miRNAs, which leaves vast spaces for studying.

### Deregulated miRNAs in invasion and metastasis

**Figure 3:**
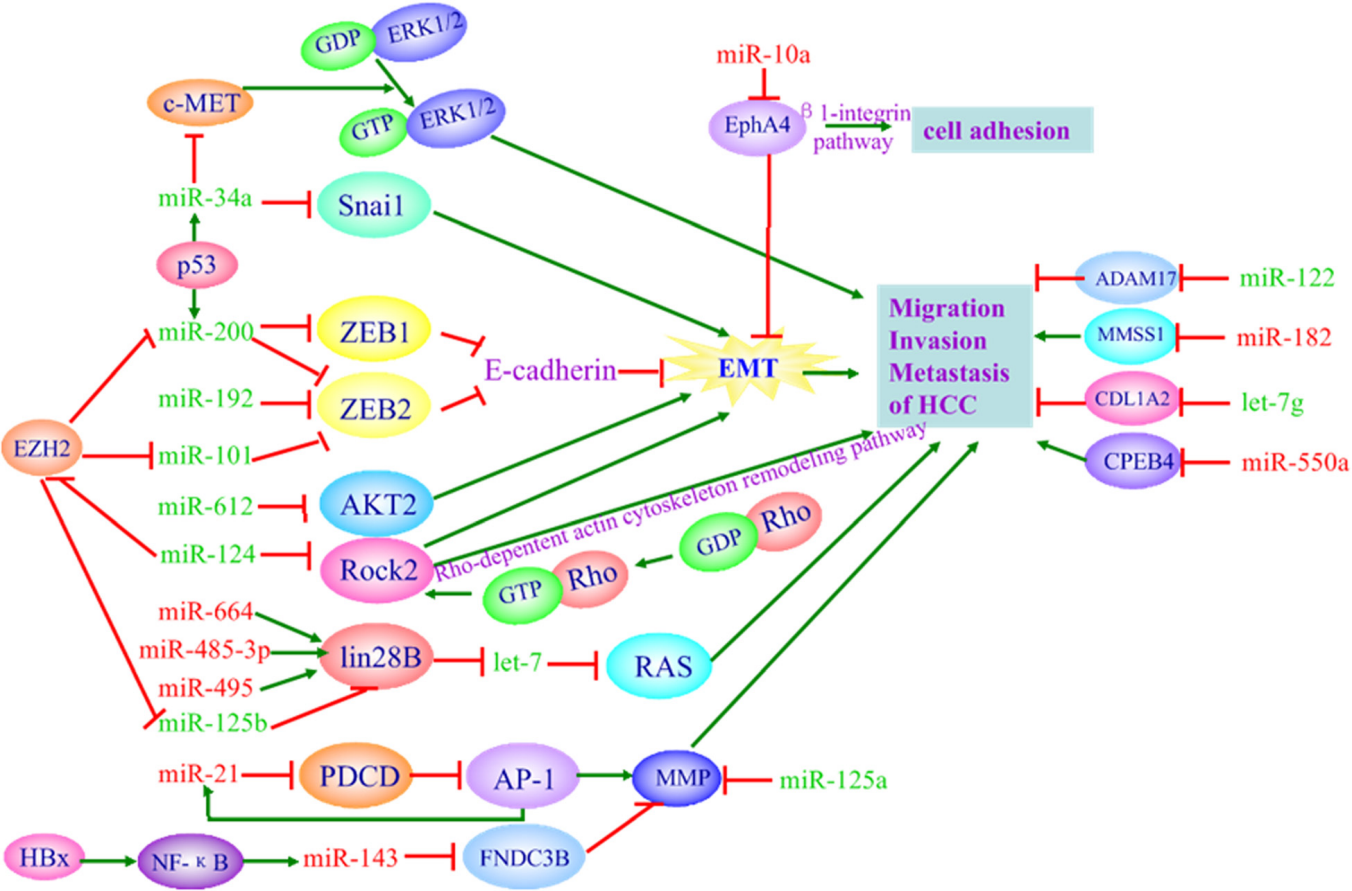
**Deregulated miRNAs in invasion and metastasis.** The expression of many miRNAs involved in HCC cell invasion and metastasis are altered and affect RAS-, RHO-, and EMT-related signaling pathways. The green miRNAs may inhibit HCC cell invasion and metastasis, and the red miRNAs may promote HCC cell invasion and metastasis.

The invasion and metastasis of tumor cells are influenced by the deregulation of components these same pathways, such as receptor tyrosine kinases (RTKs), and the downstream phosphatidyl inositol 3-kinase (PI3K)-Akt kinase signaling pathways. Overwhelming evidence indicates that different miRNAs could regulate the above pathways (see Figure 3).

Among the downstream effectors of RTK signaling pathways, Ras has shown over-expression in HCC and has been regulated by members of the let-7 family. Moreover, the downregulation of the let-7 family is often reported in HCC, suggesting a possible contribution to the upregulation of Ras [[75]]. Furthermore, miR-125b can regulate a downstream target, lin-28 homolog B (LIN28B), in HCC cells, and subsequently indirectly promote the processing of let-7. Subsequently, Ras is inhibited, which is followed by the inhibition of HCC cell migration and invasion [[76]]. However, there are still some miRNAs that could activate Ras, resulting in the promotion of HCC cell migration and invasion.

The epithelial-mesenchymal transition (EMT) is critical for HCC cell migration and invasion, and many miRNAs have been found to be directly or indirectly associated with this process [[77]]. Among them, two miRNA families, that is, the miR-200 and miR-34 families, are the most important for EMT. E-cadherin is the key component in EMT. The miR-200 family, which is upregulated by p53, can target both ZEB1 and ZEB2, two transcriptional repressors of E-cadherin [[78]]. Another family is the miR-34 family, which directly regulates SNAI1-dependent EMT [[79]]. miR-34a can also potently inhibit HCC migration and invasion by directly targeting c-Met and thereby decreasing the c-Met-induced phosphorylation of extracellular signal-regulated kinases 1 and 2 (ERK1/2), suggesting the miR-34a plays an important role in tumor migration and invasion [[80],[81]]. Apart from the above families, some miRNAs can maintain EMT. For example, miR-101 and miR-192 can repress ZEB2 and EZH2 to maintain E-cadherin expression [[78],[82]]. In addition, there are still other miRNAs that regulate the EMT process. For example, miR-612 has inhibitory effects on HCC migration, invasion and metastasis with one direct target, AKT2, through which EMT is inhibited [[83]]. The Eph tyrosine kinase receptor (EphA4) is one of target genes of miR-10a; by specifically targeting EphA4, miR-10a can act on invasion through EMT and on adhesion through the β1-integrin pathway in HCC cells [[84]]. ROCK2 (Rho-associated protein kinase 2, ROCK2) can promote EMT through a Rho-dependent actin cytoskeleton remodeling pathway to enhance the invasive and metastatic activity of HCC, and miR-124 can directly target ROCK2 and EZH2 genes to inhibit EMT, which leads to inhibition of the invasive and metastatic activity of HCC [[85]].

The following additional deregulated miRNAs are involved in the invasive and metastatic properties of HCC cells. Some of them are significantly upregulated in HCC and target some key signaling molecules that inhibit invasion and metastasis. For instance, fibronectin type III domain containing 3B (FNDC3B) regulates cell motility. In HCC, NF-kappaB can lead to the overexpression of miR-143, and miR-143 can target FNDC3B, which could promote HCC invasion and metastasis by improving cell motility [[86]]. In addition, miR-21 also plays an important role in promoting HCC migration and invasion by targeting programmed cell death 4 (PDCD4) and simultaneously upregulating downstream signaling pathway molecules, such as phospho-c-Jun, matrix metalloproteinase(MMP)-2 and MMP-9 [[87]]. Moreover, miR-550a also promotes HCC cell migration and invasion by targeting cytoplasmic polyadenylation element binding protein 4 (CPEB4) [[88]].

However, the decreased expression of many miRNAs that inhibit the invasion and metastasis of HCC are often observed in both HCC tissues and cell lines. For instance, miR-125a, which is always significantly downregulated in HCC, inhibits the metastasis of tumor cells by targeting MMP11 and vascular endothelial growth factor A (VEGF-A) [[89]]. Similarly, miR-424, which is also down-regulated in HCC, suppresses tumor cell migration and invasion through its downstream target c-Myb [[90]]. Other key signaling molecules that promote metastasis of HCC are targeted by miRNAs, including a disintegrin and metalloprotease 17 (ADAM17), metastasis suppressor 1 (MTSS1), type I collagen alpha2 (COL1A2), and other factors [[91]-[93]].

### Deregulated miRNAs in PI3K/Akt/mTOR/autophagy pathway

The phosphoinositide 3-kinase (PI3K) pathway is a very important signal transduction system that is bound up with oncogenes and various receptors to regulate many key cellular functions, such as proliferation, differentiation, migration, trafficking, apoptosis and glucose homeostasis [[94]]. The activated PI3K phosphorylates phosphatidylinositol biphosphate (PIP2) to form phosphatidylinositol triphosphatase (PIP3), thus leading to the phosphorylation and activation of Akt. The most commonly studied class I PI3K consists of a p110 catalytic subunit and a p85 regulatory subunit. p110 catalytic subunits are divided into many subunits, such as phosphoinositide 3-kinase catalytic subunit alpha (PIK3CA) and delta (PIK3CD). These catalytic subunits are regulated by miRNAs. For example, the overexpression of miR-124 leads to the downregulation of PIK3CA [[95]]; furthermore, miR-7 targets PIK3CD, mTOR, and p70S6K [[96]], both of which result in suppression of the PI3K/Akt/mTOR-signaling pathway. These facts indicate that miRNAs could act as tumor regulators by suppressing PI3K to inhibit the metastasis of HCC.

PTEN can convert PIP3 back to PIP2 and can negatively regulate the expression of PIP3. By targeting PTEN, miR-21 and miR-221 show invasive properties. miR-21 regulates various processes that promote tumor invasiveness by targeting PTEN and PDCD4 in HCC. In addition, miR-21 could target RECK, which negatively regulates MMP-9, and lead to increasing invasion [[97]]. Moreover, PTEN is one of the target genes of miR-29a and can subsequently lead to Akt phosphorylation, which regulates the migration of hepatoma cells [[98]]. However, the targeting of PTEN and AKT3 by miR-519d could contribute to hepatocarcinogenesis [[99]].

HBx has been proven to play a key role in the molecular pathogenesis of HBV-related HCC and has a close relationship with miRNAs. For example, HBx can repress miR-148a, and the latter subsequently targets hematopoietic pre-B cell leukemia transcription factor-interacting protein (HPIP), which leads to downregulation of AKT and mTOR through the AKT/ERK/FOXO4/ATF5 pathway [[100]]. HBx can also up-regulate the expression of URG11, which subsequently up-regulates β-catenin; PTEN has been shown to be targeted by miR-148a and thereby contribute to hepatocarcinogenesis [[101]]. Another study constructed a lentivirus vector and performed dual-luciferase reporter assays to confirm that miR-29a could target SPARC and thereby both inhibit the phosphorylation of SPARC/AKT/mTOR and HCC growth [[102]].

mTORC1 and mTORC2 are two different complexes of mTOR. AKT activates mTORC1, while mTORC2 activates AKT. There are many miRNAs that target mTOR. For example, miR-99a, which was found to be significantly decreased in HCC, could directly target insulin-like growth factor 1 receptor (IGF-1R) and mTOR, suggesting that miR-99a is a promising tumor suppressor for HCC [[103]]. Apart from miR-99a, miR-199a-3p also targets mTOR in HCC cells [[97]]. In addition, mTOR could also be regulated by miRNAs indirectly. For example, DNA damage-inducible transcript 4 (DDIT4), a modulator of the mTOR pathway, has been identified as a target of miR-221; thus, miR-221 can regulate the mTOR pathway indirectly [[104]].

Activated mTORC1 downregulates autophagy. The hallmark of autophagy is the transition of LC3-I to its lipidated form (LC3-II), which requires the help of autophagy-related-gene 7 (Atg 7) [[95]]. To date, two miRNAs were demonstrated to be related to the autophagy process of HCC. One is miR-375, which inhibits autophagy by targeting ATG7 and weakens the viability of HCC under hypoxic conditions [[105],[106]]. Another one is miR-199-5p, whose downregulation ensures autophagy by targeting ATG7. Often, HCC patients have a low level of miR-199a-5p after treatment with cisplatin-based chemotherapy. Cisplatin treatment also results in decreased miR-199a-5p levels in human HCC cell lines. miR-199a-5p/autophagy signaling represents a novel pathway that regulates chemoresistance and thus offers a new target for the cisplatin-based chemotherapy of HCC [[107]].

The insulin receptor substrate1 (IRS1) protein is a key mediator of insulin-like growth factor (IGF) signaling which regulates a variety of cellular processes including growth, differentiation, survival and metabolism. IRS1 is targeted by miRNA-145 and they are negatively correlated in HCC. Thus, the dysregulation of miR-145 may contribute to a potential molecular mechanism of carcinogenesis in HCC [[108]].

## Conclusions

Ten years of research on this class of tiny non-coding RNAs has provided significant understanding, confirming that miRNAs have emerged as novel players in cell growth, differentiation, proliferation, apoptosis, inflammation and tumorigenesis. There is sufficient evidence to indicate that the specific expression of miRNAs affects the occurrence and development of tumors and may be associated with pathobiology and clinical features. Moreover, functional and target connection studies on dysregulated miRNAs in HCC and their key enzymes have provided us a more comprehensive understanding of their roles in oncogenic signaling pathways. This knowledge could clarify the molecular pathogenesis and the occurrence and development of HCC. All the above will prompt us to find new diagnostic markers and potential methods to treat HCC. Nevertheless, translating the research data on the mechanistic cause of miRNA dysregulation to applications for clinical practice will require significant work and will be an important field for HCC study.

## Competing interests

The authors declare that they have no competing interests.

## Authors’ contributions

RC and GM wrote the first version of the manuscript, and incorporated all the contributions from the coauthors. All authors read and approved the final manuscript.
